# Analyzing special needs reports for children: sociodemographic trends, diagnoses, and support areas over five years (2019–2024)

**DOI:** 10.3389/fped.2024.1472553

**Published:** 2025-01-20

**Authors:** Semih Canpolat, Mehmet Emin Parlak, Esra Kurt Canpolat, Erdogan Oz

**Affiliations:** ^1^ Department of Child Health and Diseases, Batman Provincial Directorate of Health, Batman, Türkiye; ^2^ Department of Child Health and Diseases, University of Health Sciences, Mersin City Education and Research Hospital, Mersin, Türkiye; ^3^ Department of Family Practice, University of Health Sciences, Gazi Yasargil Education and Research Hospital, Diyarbarkir, Türkiye; ^4^ Department of Family Practice, Prof. Dr. Cemil Tascioglu City Hospital, Istanbul, Türkiye

**Keywords:** SNRC, special needs, health board, child, adolescent

## Abstract

**Objective:**

This study aims to evaluate the special needs reports for children (SNRC) in terms of sociodemographic characteristics, diagnoses, and areas of special needs in the five years from 2019, when special health boards for children were established with the new diagnostic and evaluation criteria.

**Methods:**

This descriptive study was conducted by retrospectively reviewing the board evaluation reports of children aged 0–18 years who applied to Batman Training and Research Hospital SNRC Health Board between March 2019 and March 2024. The study included 420 children for whom all the data in the evaluation reports could be accessed. All data of the participants were collected and analyzed through the Hospital Information Management System (HIMS).

**Results:**

The mean age at the time of health board admission was 7.41 years with 58.3% boys and 41.7% girls. When the level of special needs was analyzed, the highest rate of 47.1% was found to have Special Condition Requirement Needs (SCRN). The most common reason for application in both boys (68.6%) and girls (64%) was to benefit from Disability Rights. In terms of the distribution of disease areas, the highest rate was in the Cognitive Development Area with 36.2%, the second highest rate was in the Movement Development Area with 27.1%, and the lowest rates were in the Genitourinary System - Surgery Area with 0.2% and in the Hematology-Oncology Area with 0.2%. When the areas of Specific Learning Disabilities (SLD) were analyzed, it was determined that the highest rate was in the area of rehabilitation/early support (intervention) requirement to support cognitive functions with 43.1%. When the special needs levels were analyzed according to the disease areas, the highest rate was found in 13 areas except for the Nephrology area and Genitourinary System-Surgery Area was SCRN. In Nephrology, the rates were equally shared between SCRN and Significant special needs (SSPD) at 50%, while the highest rate in the Genitourinary System-Surgery Field was Special needs (SEN) with 60.6%.

**Conclusion:**

Our research emphasizes the crucial role of SNRC health board reports in tackling the pressing challenges faced by children and their families. Through harnessing these reports, we can make significant progress in identifying and supporting children with special needs, ultimately enhancing their quality of life. Our findings emphasize the influence of gender and the typical age of 7–8 years for initial evaluation. Looking ahead, it is vital to develop comprehensive strategies to raise community awareness and equip healthcare professionals with the necessary tools to optimize support systems. Through ongoing analysis and adaptation, these efforts hold the promise of fostering a more inclusive and supportive environment for children with diverse needs.

## 1 Introduction

Special need is a state of biopsychosocial inadequacy manifested by impairment and limitation in physical, mental, psychological, and social abilities due to various reasons that occur congenitally or acquired (1). Individuals with special needs have difficulty in performing and maintaining ordinary daily activities without assistance and in adapting to social life at an acceptable level (2). Children with special needs are children who have a higher number and intensity of needs compared to their peers due to their physical, physiological, mental, or behavioral differences. The inadequacies of these children in their adaptation skills and in meeting daily needs cause them to need regular counseling, protection, care, or rehabilitation services (3).

According to World Health Organization data, 15.3% of the world population in all age groups and 5.2% of children under the age of 14 are disabled. According to the Turkey Disability Survey, 12.29% of the population in Turkey is “disabled”. Under 19 years of age, the rate of orthopedic, visual, hearing, speech, and mental disability was 3.5%, while the rate of disability was 8.8% when other internal and psychiatric diseases were included (1, 4).

While adults and children were previously evaluated by the same health board in Turkey, special health boards for children were established in hospitals with the regulation that entered into force on February 20, 2019. In line with this regulation, which aims to determine the special needs status of children instead of the disability rate, the Special Needs Report for Children (SNRC) is evaluated and decided by these newly established boards (5).

For children with special needs to benefit from some economic rights such as health, care, special education, and tax reduction, it is necessary to apply to SNRC health boards with the guidance of physicians, teachers, rehabilitation centers, or with the direct request of families (4). The gains to be obtained with the result of the health committee can enable children with special needs and their families to be more involved in normal life. In this way, the birthright of every individual to be in social life can be fully maintained for children with special needs (6). In addition, today, laws, practices, and regulations for individuals with special needs are considered as one of the development indicators of countries (2).

This study aims to examine SNRC reports in the five years from 2019, when special health boards for children were established with the new diagnostic and evaluation criteria, and to draw conclusions that can guide both legislators and health professionals regarding special needs. Thanks to this awareness that will be spread throughout society, it may be possible for individuals with special needs to maintain their lives most easily and adapt to the social and cultural environment.

## 2 Materials and methods

This descriptive study was conducted by retrospectively screening the board evaluation reports of children aged 0–18 years who applied to the Batman Training and Research Hospital SNRC Health Board between March 2019 and March 2024. Approval for the study was obtained from Batman Training and Research Hospital Scientific Research Ethics Committee with the decision dated January 25, 2024, and numbered 375.

All data of the participants were collected and analyzed through the Hospital Information Management System (HIMS).

SNRC Health Board has 6 permanent members, including the chairman, who is a specialist in Pediatrics, and the authorized physician of SNRC. The branches of the board are Pediatrics, Child and Adolescent Mental Health and Diseases, Ophthalmology, Physical Medicine and Rehabilitation, and Ear, Nose and Throat Diseases.

Levels of special needs:

• No special requirements
• Special needs (SEN): 20–39
• Mild special needs: 40–49
• Moderate special needs: 50–59
• Advanced special needs: 60–69
• Very severe special needs: 70%–79% (considered severely disabled)
• Significant special needs (SSPD): 80%–89% (considered to be severely disabled)
• Special Condition Requirement (SCRN): 90%–99% (considered to be severely disabled)

Reasons for Application:

• Benefiting from disability rights
• To benefit from home care services
• Benefiting from special education services
• For children, a medical board report stating the situation due to terrorism, accident, and injury
• For caregiver salary (benefiting from Law No. 2828)

Disease Areas:

• Cognitive Development Area
• Child and Adolescent Psychiatry
• Speech-Language-Communication Development
• Endocrine System Area
• Genitourinary System - Surgical Area
• Visual Function Area
• Movement Development Area
• Hematology-Oncology Area
• Hearing Function - Ear, Nose, and Throat Area
• Hereditary-Natal Diseases Area
• Heart, Circulatory System Area
• Metabolism Area
• Nephrology Area
• Digestive System Area
• Nervous System Area
• Burns Area

In addition to the child's disability, the Board decided on the level of special needs and the reasons for application. Diagnoses were determined according to the ICD-10 code. Psychiatric diagnoses were made by child and adolescent psychiatrists according to DSM-5. In children older than 6 years of age, the Wechsler Intelligence Scale for Children (Wechsler Intelligence Scale for Children-R) was administered by experienced psychologists to determine the level of intelligence, and the Specific Learning Disability (SLD) battery was administered in cases where Specific Learning Disorder (SLD) was considered. In children younger than six years of age, Denver II or Ankara Developmental Screening Inventory (ADSI) administered by child development specialists was used (1).

SLD is categorized as follows:

• SLD1: Need for rehabilitation/early support (intervention) to support cognitive functions
• SLD2: Need for physiotherapy, occupational therapy, rehabilitation
• SLD3: Need for devices, orthoses, prostheses, wheelchairs, and other equipment
• SLD4: Speech and language therapy/rehabilitation requirement
• SLD5: Need for therapy/rehabilitation for hearing impairment/loss
• SLD6: Therapy/rehabilitation requirement for visual function limitation/loss
• SLD7: Therapy/rehabilitation needs for autism spectrum disorder
• SLD8: Therapy/rehabilitation requirement for specific learning disabilities
• SLD9: Need for rehabilitation at home or in hospital
• SLD10: Other

Inclusion Criteria:

• Age between 0 and 18 years
• Completion of application to SNRC
• Accessibility of complete file information

Exclusion Criteria:

• Patients with incomplete information in their files are excluded.

### 2.1 Statistical analysis

The data in the study were analyzed using the SPSS program (Statistical Package for Social Sciences) 25.0. Continuous data were expressed as mean ± standard deviation, median, minimum, and maximum. Pearson's Chi-Square test was used to compare categorical data. Since the study was descriptive, no further comparisons were made. Statistical significance was accepted as *p* < 0.05.

## 3 Results

The reports of 453 children who applied to the SNRC Health Board, which was established specifically for children within five years, were analyzed. A total of 420 (92.7%) children aged 0–18 years with complete file information were included in the study. The 33 (7.3%) children for whom all the data in the evaluation reports could not be accessed were excluded from the study. Of the 420 patients included in the study, 58.3% were boys and 41.7% were girls. The mean age of the patients was 7.41 years (±4.84 SD), the median age was 7 years, the minimum age was 0 and the maximum age was 17 years. When the special needs levels of the children were analyzed, it was seen that the highest rate was 47.1% in the “Special Condition Requirement (SCRN): 90%–99% (considered to be severely disabled)” group, followed by 26.7% in the “Special needs (SCRN): 20%–39%” and 8.3% in the “Moderate special needs: 50%–59%” groups (Table 1).

**Table 1:** General distribution.

		*n*	%
Gender	Male	245	58.3
	Girl	175	41.7
	Total	420	100,0
Special requirement level	No special requirements	25	6.0
	Special needs (SEN): 20–39	112	26.7
	Mild special needs: 40–49	10	2.4
	Moderate special needs: 50–59	35	8.3
	Advanced special needs: 60–69	6	1.4
	Very severe special needs: 70%–79% (considered severely disabled)	17	4.0
	Significant special needs (SSPD): 80%–89% (considered to be severely disabled)	17	4.0
	Special Condition Requirement (SCRN): 90%–99% (considered to be severely disabled)	198	47.1
	Total	420	100.0
Application causes	Benefiting from disability rights	280	66.7
	To benefit from home care services	112	26.7
	Benefiting from special education services	26	6.2
	For children, a medical board report stating the situation due to terrorism, accident, and injury	1	0.2
	For caregiver salary (benefiting from Law No. 2828)	1	0.2
	Total	420	100.0
Age	Mean - SD	7.41 (4.84)	
	Min-Maks-Median	0–17–7	

When the special needs levels of the children included in the study were analyzed separately according to gender, it was seen that the highest rate of 44.9% in boys was “Special Condition Requirement (SCR): 90%–99% (considered to be severely disabled)”, followed by “Special needs (SCR): 20%–39%” with 31.4% and “Moderate special needs: 50%–59%” with 6.9%. When the distribution of girls was analyzed, it was seen that the highest rate was “Special Condition Requirement (SCRN): 90%–99% (considered to be severely disabled)” with 50.3%, followed by “Special needs (SCRN): 20%–39%” with 20% and “Moderate special needs: 50%–59%” with 10.3% (Table 2).

**Table 2:** Distribution of special needs levels.

	Male		Girl		*X* ^2^	*p*
	*n*	%	*n*	%		
No special Total requirements	14	% 5.7	11	% 6.3	8.130	0.321
Special needs (SEN): 20–39	77	% 31.4	35	% 20.0		
Mild special needs: 40–49	6	% 2.4	4	% 2.3		
Moderate special needs: 50–59	17	% 6.9	18	% 10.3		
Advanced special needs: 60–69	3	% 1.2	3	% 1.7		
Very severe special needs: 70%–79% (considered severely disabled)	8	% 3.3	9	% 5.1		
Significant special needs (SSPD): 80%–89% (considered to be severely disabled)	10	% 4.1	7	% 4.0		
Special Condition Requirement (SCRN): 90%–99% (considered to be severely disabled)	110	% 44.9	88	% 50.3		
Total	245	% 100.0	175	% 100.0		

When the reasons for the application of the children included in the study were analyzed separately by gender, it was seen that the highest rate of both boys (68.6%) and girls (64%) was “To Benefit from Disability Rights” (Table 3).

**Table 3:** Distribution of reasons for application.

	Male		Girl		*X* ^2^	*p*
	*n*	%	*n*	%		
Benefiting from disability rights	168	% 68.6	112	% 64.0	5.680	0.224
To benefit from home care services	58	% 23.7	54	% 30.9		
Benefiting from special education services	18	% 7.3	8	% 4.6		
For children, a medical board report stating the situation due to terrorism, accident, and injury	1	% 0.4	0	% 0		
For caregiver salary (benefiting from Law No. 2828)	0	% 0	1	% 0.6		
Total	245	% 100.0	175	% 100.0		

When the distribution of disease areas was analyzed, it was determined that the highest rate was observed in the Cognitive Development Area (36.2%), while the lowest rates were observed in the Genitourinary System - Surgery Area (0.2%) and Hematology-Oncology Area (0.2%) (Table 4).

**Table 4:** Distribution of disease areas.

		*n*	%
Cognitive development area	No	268	63.8
	There is	152	36.2
	Total	420	100.0
Child and adolescent psychiatry	No	358	85.2
	There is	62	14.8
	Total	420	100.0
Speech-language-communication development	No	387	92.1
	There is	33	7.9
	Total	420	100.0
Endocrine system area	No	390	92.9
	There is	30	7.1
	Total	420	100.0
Genitourinary system - surgical area	No	419	99.8
	There is	1	0.2
	Total	420	100.0
Visual function area	No	392	93.3
	There is	28	6.7
	Total	420	100.0
Movement development area	No	306	72.9
	There is	114	27.1
	Total	420	100.0
Hematology-oncology area	No	419	99.8
	There is	1	0.2
	Total	420	100.0
Hearing function - ear, nose, and throat area	No	395	94.0
	There is	25	6.0
	Total	420	100.0
Hereditary-natal diseases area	No	360	85.7
	There is	60	14.3
	Total	420	100.0
Heart, circulatory system area	No	416	99.0
	There is	4	1.0
	Total	420	100.0
Metabolism area	No	418	99.5
	There is	2	0.5
	Total	420	100.0
Nephrology area	No	416	99.0
	There is	4	1.0
	Total	420	100.0
Digestive system area	No	401	95.5
	There is	19	4.5
	Total	420	100.0
Nervous system area	No	331	78.8
	There is	89	21.2
	Total	420	100.0
Burns area	No	408	97.1
	There is	12	2.9
	Total	420	100.0

The distribution of the patients participating in the study in the areas of SLD is shown in the table. According to this; it was observed that the highest rate was in the field of SLD1 (43.1%). It was determined that there was no patient in the area of SLD10 (Table 5).

**Table 5:** Distribution of areas of specific learning disabilities.

		*n*	%
SLD1	No	239	56.9%
	There is	181	43.1%
	Total	420	100.0%
SLD2	No	244	58.1%
	There is	176	41.9%
	Total	420	100.0%
SLD3	No	313	74.5%
	There is	107	25.5%
	Total	420	100.0%
SLD4	No	370	88.1%
	There is	50	11.9%
	Total	420	100.0%
SLD5	No	398	94.8%
	There is	22	5.2%
	Total	420	100.0%
SLD6	No	389	92.6%
	There is	31	7.4%
	Total	420	100.0%
SLD7	No	362	86.2%
	There is	58	13.8%
	Total	420	100.0%
SLD8	No	397	94.5%
	There is	23	5.5%
	Total	420	100.0%
SLD9	No	340	81.0%
	There is	80	19.0%
	Total	420	100.0%
SLD10	No	420	100.0%
	There is	0	0.0%
	Total	420	100.0%

SLD, specific learning disability.

Pearson's Chi-Square test was performed to determine whether there was a statistical difference between the gender of the participants and their SLD status. According to the test results; no statistically significant relationship was found between gender variable and SLD1 (*p* = 0.188), SLD2 (*p* = 0.082), SLD4 (*p* = 0.959), SLD5 (*p* = 0.139), SLD6 (*p* = 0.875), SLD8 (*p* = 0.404) and SLD9 (*p* = 0.153). On the other hand, a statistically significant relationship was found between the gender variable and SLD3 (*p* = 0.002) and SLD7 (*p* = 0.013). While the “None” group was higher in boys than the expected value and the “Present” group was higher in girls than the expected value for the SLD3 variable, it was concluded that the “None” group was higher in girls than the expected value and the “Present” group was higher in boys than the expected value for the SLD7 variable (Table 6).

**Table 6:** Comparison of specific learning disabilities by gender.

			Male	Girl	Total	*p*
SLD1	No	Number	146	93	239	0.188
		Percentage	61.1%	38.9%	100.0%	
	There is	Number	99	82	181	
		Percentage	54.7%	45.3%	100.0%	
SLD2	No	Number	151	93	244	0.082
		Percentage	61.9%	38.1%	100.0%	
	There is	Number	94	82	176	
		Percentage	53.4%	46.6%	100.0%	
SLD3	No	Number	196	117	313	0.002
		Percentage	62.6%	37.4%	100.0%	
	There is	Number	49	58	107	
		Percentage	45.8%	54.2%	100.0%	
SLD4	No	Number	216	154	370	0.959
		Percentage	58.4%	41.6%	100.0%	
	There is	Number	29	21	50	
		Percentage	58.0%	42.0%	100.0%	
SLD5	No	Number	236	162	398	0.139
		Percentage	59.3%	40.7%	100.0%	
	There is	Number	9	13	22	
		Percentage	40.9%	59.1%	100.0%	
SLD6	No	Number	226	163	389	0.875
		Percentage	58.1%	41.9%	100.0%	
	There is	Number	19	12	31	
		Percentage	61.3%	38.7%	100.0%	
SLD7	No	Number	202	160	362	0.013
		Percentage	55.8%	44.2%	100.0%	
	There is	Number	43	15	58	
		Percentage	74.1%	25.9%	100.0%	
SLD8	No	Number	234	163	397	0.404
		Percentage	58.9%	41.1%	100.0%	
	There is	Number	11	12	23	
		Percentage	47.8%	52.2%	100.0%	
SLD9	No	Number	204	136	340	0.153
		Percentage	60.0%	40.0%	100.0%	
	There is	Number	41	39	80	
		Percentage	51.2%	48.8%	100.0%	

Special Needs Levels according to disease areas are shown in Table 7.

**Table 7:** Special needs levels by disease area (%).

Fields	No special requirements	Special needs (SCF): 20–39	Mild special needs: 40–49	Moderate special needs:%50–59	Advanced special needs: 60–69	Very Severe special needs: 70–79 (Considered to be Severely Disabled)	Significant special needs (SSN): 80–89 (Considered to be Severely Disabled)	Special condition requirement (SCRR): 90–99 (Considered to be Severely Disabled)
Cognitive development area	4.0%	28.0%	4.0%	0.0%	0.0%	4.0%	0.0%	60.0%
Child and adolescent psychiatry	3.3%	8.3%	3.3%	6.7%	1.7%	5.0%	3.3%	68.3%
Speech-language-communication development	0.0%	19.7%	2.6%	14.5%	1.3%	6.6%	3.9%	51.3%
Endocrine system area	0.0%	33.9%	1.6%	3.2%	0.0%	1.6%	4.8%	54.8%
Genitourinary system - surgical area	0.0%	60.6%	3.0%	3.0%	3.0%	0.0%	3.0%	27.3%
Visual function area	0.0%	20.0%	0.0%	0.0%	3.3%	0.0%	13.3%	63.3%
Movement development area	0.0%	0.0%	0.0%	0.0%	0.0%	0.0%	0.0%	100.0%
Hematology-oncology area	0.0%	35.7%	7.1%	3.6%	7.1%	3.6%	0.0%	42.9%
Hearing function – ear, nose, and throat area	0.0%	25.4%	2.6%	8.8%	2.6%	2.6%	5.3%	52.6%
Hereditary-natal diseases area	0.0%	0.0%	0.0%	0.0%	0.0%	0.0%	0.0%	100.0%
Metabolism area	0.0%	0.0%	0.0%	0.0%	0.0%	0.0%	0.0%	100.0%
Nephrology area	0.0%	0.0%	0.0%	0.0%	0.0%	0.0%	50.0%	50.0%
Digestive system area	5.3%	15.8%	0.0%	10.5%	0.0%	5.3%	15.8%	47.4%
Nervous system area	3.4%	18.0%	2.2%	7.9%	3.4%	5.6%	4.5%	55.1%
Burns area	0.0%	16.7%	8.3%	8.3%	8.3%	16.7%	8.3%	33.3%

## 4 Discussion

The problems faced by children with special needs are problems that not only children and their families but also the whole of society must face and find solutions to. The reports from SNRC health boards can help children and therefore their families to achieve some gains, which in turn can reduce and facilitate the problems. For this reason, studies like our study in which the reports of SNRC health boards are analyzed may be guiding for a solution (4, 7).

Of the 420 patients in our study, 58.3% were boys and 41.7% were girls. In parallel with our study, in many studies on SNRC, it was found that the rate of boys applying to the board was significantly higher than the rate of girls (1–4, 6–9). Some diseases that may cause special needs such as specific learning disorders, autism spectrum disorder, hemophilia, and Duchenne muscular dystrophy are more common in boys. Boys are also more likely to be exposed to negative environmental factors. A higher social awareness of behavioral and developmental disorders in boys may lead to easier identification of special needs. Therefore, gender should be considered an important factor in the identification and support of special needs.

Similar to our study in which the mean age was found to be 7.41 years, in many studies in Turkey in which SNRC reports were analyzed, it was found that the mean age of children who applied to the health board was 7–8 years (3, 7, 9, 10). Most families may not recognize the status of their children with mild special needs in the preschool period. At the age of 6–7, which is the age of school entry, families who suspect that their child may be an individual with special needs due to academic inadequacy take their children to the doctor. Sometimes the teacher may detect the difference in the child and direct the families to apply to the hospital. All these reasons explain why the average age of application to the SNRC health committee in most studies is around the age of seven. Therefore, for SNRC health boards to become more functional, arrangements should be planned to prioritize the needs of this age group. In addition, implementing projects to raise awareness among families in the preschool period and encouraging family physicians and family health workers to identify children with special needs may pave the way for early identification of children with special needs.

When the special needs levels of the children included in our study were examined, SCRN (90%–99%) was found with the highest rate of 47.1% by most studies in the literature (1, 6, 8, 9, 11). In the study conducted by Güller & Yaylacı, the highest rate of SEN was found to be 45.5% and the second highest rate of SCRN was found to be 27.7% (4). In line with some studies, when the distribution of special needs levels was examined in our study, it was observed that there was no significant difference between boys and girls (7–9). However, in some other studies, it was reported that there was a difference between male and female gender (1). The fact that the studies yielded different results reveals that the methods and criteria used in determining the levels of special needs should be re-evaluated.

When the distribution of the reasons for application was analyzed, no significant difference was found between boys and girls in our study and it was observed that the most common reason for application was to benefit from disability rights with 68.6% in boys and 64% in girls. In a study conducted by Kumbul et al. similar to our study, it was found that the most common reason for application was to benefit from disability rights (9). Unlike these results, in a study conducted by Kayhan & Öztürk, the most common reason for application was special education (52.4%) (8). The reason for this difference in the studies may be the difference in the perspective of special needs in the places where the studies were conducted, the fact that the primary need in children and families may vary according to sociocultural characteristics, the age distribution of the participants not overlapping, the different physical and environmental structure in the place of residence, and the lack of consensus among the physicians in the health committee on diagnostic criteria.

When the distribution of disease areas was examined in our study, it was seen that the highest rate was in the Cognitive Development Area with 36.2%, the second highest rate was in the Movement Development Area with 27.1%, and the lowest rates were in the Genitourinary System - Surgery Area with 0.2% and in the Hematology-Oncology Area with 0.2%. In the study conducted by Güller & Yaylacı, the highest rate of 44.7% was again in the Cognitive Development Field, while the second highest rate of 41.9% was in the Child and Adolescent Psychiatry Field (4). In the study conducted by Yıldız&Tarakçıoğlu, it was found that the highest rate was in the field of Child and Adolescent Psychiatry with 41.1% (2). These findings show that special needs can be seen in a wide range and at very different rates. Therefore, it is important to consider individual needs in the assessment and treatment of special needs. In addition, it is also important to closely monitor the Cognitive Developmental Domain.

In the present study, when the distribution of SLD areas was analyzed, SLD1 was found in 43.1%, SLD2 in 41.9%, SLD3 in 25.5%, SLD4 in 11.9%, SLD5 in 5.2%, SLD6 in 7.4%, SLD7 in 13.8%, SLD8 in 5.5%, and SLD9 in 19%. There were no children in the area of SLD10 (Table 5). This distribution shows that SLD1, SLD2, and SLD3 are at the forefront. This situation is important for both preventive medicine and diagnosis, treatment, and follow-up of children. In short, our study reveals that children should be followed up more closely in terms of certain needs. The way to achieve this is to educate both families and health professionals and to develop medical and administrative strategies that will lead to awareness in the community.

In the present study, no statistically significant relationship was found between boys and girls in terms of SLD1 (*p* = 0.188), SLD2 (*p* = 0.082), SLD4 (*p* = 0.959), SLD5 (*p* = 0.139), SLD6 (*p* = 0.875), SLD8 (*p* = 0.404) and SLD9 (*p* = 0.153). On the other hand, while SLG3 was higher in girls (54.2%), SLG7 was higher in boys (74.1%) (Table 6). This result reveals that boys and girls should be evaluated separately in the evaluation of SNRC in planning for both prevention treatment and rehabilitation.

In the present study, when the levels of special needs according to the disease areas were analyzed, the highest rate was determined as SCRN in 13 areas except for the field of Nephrology and Genitourinary Systems. In the field of nephrology, the rates were equally shared between SCRN and SSPD at 50%, while in the field of Genitourinary system surgery, the highest rate was found to be SEN with 60.6% (Table 7) In a study conducted by Kumbul et al. in Isparta in 2020, it was found that a different need had a higher rate in almost every field, which was very different from our study (9). These differences may be due to the difference in the geographical region where the study was conducted, as well as the change in the profile of patients admitted to the hospital due to the impact of the COVID-19 pandemic and the change in the expectations of families to apply to the health committee. The identification and management of special needs can be influenced by many factors such as geographical healthcare infrastructure and sociocultural dynamics. This also illustrates the difficulty of standardizing special needs for adults and children. In other words, the most important outcome of our study is that individualized health services are vital.

Many activities and programs are recommended for children and young people with special needs to be more active in social life and to increase the self-confidence of themselves and their families (12). However, the most important point here is to pave the way for children to be diagnosed correctly and to benefit from their legal rights in the maximum way. The primary way to achieve this is to develop strategies to ensure that the SNRC health boards are structured in a faster, more objective, and more inclusive manner through future studies.

The biggest limitation of the study is that it is not known how many of the families of the children included in the study objected to the report and whether the results of the objected reports changed. Another limitation of the study is that the physicians in the health committee changed within 5 years, and accordingly, the patients included in the study may have been evaluated by physicians with different approaches. In addition, since Batman, where the study was conducted, is located in the Southeastern Anatolia region of Turkey, the results may vary too much to be generalized to other provinces with significant geographical and cultural differences. The advantageous aspect of the study is that it covers the entire five-year period from the first day of the regulation of the SNRC.

## 5 Conclusion

The study shows that SNRC health board reports can mitigate crucial problems by providing important benefits for children and their families. In this context, analyzing SNRC reports can serve as a guide in determining solutions. The findings of our study revealed that gender is an important factor in the identification and support of special needs, and the age of presentation is usually 7–8 years. In addition, it is of great importance to develop strategies to raise awareness in the community and to train health professionals. Further research and continuous monitoring are crucial for improving outcomes and support systems for these children and their families.

## Data Availability

The raw data supporting the conclusions of this article will be made available by the authors, without undue reservation.
